# Protective effect of *Monarda didymaL.* essential oil and its main component thymol on learning and memory impairment in aging mice

**DOI:** 10.3389/fphar.2022.992269

**Published:** 2022-08-29

**Authors:** Yingxue Guo, Yan Qu, Wenpeng Li, Hongkuan Shen, Jiwen Cui, Jiguang Liu, Jinlian Li, Dongmei Wu

**Affiliations:** ^1^ Key Laboratory of Microecology-Immune Regulatory Network and Related Diseases, School of Basic Medicine, Jiamusi University, Jiamusi, Heilongjiang, China; ^2^ College of Pharmacy, Jiamusi University, Jiamusi, Heilongjiang, China; ^3^ College of Jiamusi, Heilongjiang University of Chinese Medicine, Jiamusi, Heilongjiang, China; ^4^ School of Stomatology, Jiamusi University, Jiamusi, Heilongjiang, China; ^5^ Jiamusi Inspection and Testing Center, Jiamusi, Heilongjiang, China

**Keywords:** Monarda didymaL. essential oil, Thymol, learning and memory impairment, aging, Nrf2/HO-1 and MAPK pathways

## Abstract

The aging process of human beings is accompanied by the decline of learning and memory ability and progressive decline of brain function, which induces Alzheimer’s Disease (AD) in serious cases and seriously affects the quality of patient’s life. In recent years, more and more studies have found that natural plant antioxidants can help to improve the learning and memory impairment, reduce oxidative stress injury and aging lesions in tissues. This study aimed to investigate the effect of *Monarda didymaL.* essential oil and its main component thymol on learning and memory impairment in D-galactose-induced aging mice and its molecular mechanism. The composition of *Monarda didymaL.* essential oil was analyzed by Gas Chromatography-Mass Spectrometer (GC-MS). A mouse aging model was established by the subcutaneous injection of D-galactose in mice. The behavior changes of the mice were observed by feeding the model mice with essential oil, thymol and donepezil, and the histopathological changes of the hippocampus were observed by HE staining. And the changes of acetylcholinesterase (AchE), superoxide dismutase (SOD) and glutathione peroxidase (GSH-PX) activities, and the content of malondialdehyde (MDA) in hippocampal tissues were detected by corresponding kits. The expression of mitogen activated protein kinase (MAPK) and nuclear factor E2 related factor 2 (Nrf2) pathways related proteins were detected by western blot. Animal experimental results showed that compared with model group, the above indexes in *Monarda didymaL.* essential oil and thymol groups improved significantly in a dose-dependent manner. *Monarda didymaL.* essential oil and its main active component thymol can improve the learning and memory impairment of aging mice to some extent, and Nrf2 and MAPK pathways may be involved in its action process.

## 1 Introduction

With the aggravation of aging, the number of patients with neurodegenerative diseases such as AD is increasing gradually. It is estimated that by 2050, there will be more than 100 million AD patients in the world, which will seriously affect the life quality and families of patients and increase the economic and social burden (Masters., 2020). In recent years, it has been pointed out that oxidative stress plays an important role in aging, learning and memory impairment and the onset of AD, which may be the earliest pathological feature of brain injury in AD patients (ForetDo Carmo et al., 2020). Moreover, it can interact with the processes of amyloid β-protein (Aβ) deposition (Cheignon et al., 2018), inflammatory reaction (Wilkinson and Landrenth, 2006), Tau protein phosphorylation (Alavi Naini and Soussi-Yanicostas, 2015), and activate multiple signaling pathways such as MAPK, mTOR, PI3K/AKT, which continuously aggravate the neuronal damage in patients and eventually cause learning and memory impairment or even dementia in patients. Therefore, it is urgent to find effective interventions to delay neural aging and improve learning, memory, and cognitive function of the prevention and treatment of neurodegenerative disease, which is also a major challenge in the field of geriatrics.

Natural products of medicinal value have attracted increasing attention due to the serious side effects usually caused by chemical drugs. In recent years, the medicinal value of natural active antioxidant components has also been confirmed gradually. Various natural phytoactive components with antioxidant functions such as Resveratrol (Gueguen et al., 2015), Curcumin (Banji et al., 2014), ginkgolide (Shao et al., 2021) and others have also been shown to have therapeutic value in degenerative neurological diseases, not only reducing Aβ-protein, but also has some effects on apoptosis, autophagy, neuroinflammation, oxidative stress, and mitochondrial function, among which the antioxidant function may serve as a potential basis for the treatment of neurodegenerative disease. The antioxidant effects of natural plant essential oils have also been developed and utilized. Many essential oils such as Rimulus cinnamon (Lim et al., 2001), Lavandula angustifolia Mill (Xu et al., 2017). And Alpinia oxyphylla (Xu et al., 2020) have been proved to display nervous system activity and can effectively improve the learning and cognitive impairment of mice caused by scopolamine. However, the relevant research is still in the basic stage. The development of more natural plant ingredients with antioxidant activity can provide new options for the research and development of new drugs for the treatment of neurodegenerative diseases.

*Monarda didymaL.* is a plant of *Monarda didymaL.* of Labiate originated from America. Studies have shown that the *Monarda didymaL.* essential oil had strong anti-inflammatory, anti-bacteria and anti-oxidation effects, and thymol was the main component (Ricci et al., 2017). Thymol has been proved to be a potent antioxidant substance in the previous study, it showed that thymol seemed to play a positive role in the expression and maturation of BDNF in the hippocampus, with a significant anti-depressant-like effect (Capibaribe et al., 2019). Thymol can improve brain insulin resistance and up-regulate the expression of Nrf2 and HO-1 in Nrf2/HO-1 pathway to improve the cognitive impairment of mice induced by high-fat diet (FangFang et al., 2016). It was reported that thymol had application potential in the anti-depression and cognitive impairment and other cranial nerves. However, the protective effects of *Monarda didymaL.* essential oil and its main component thymol on neuroprotection such as learning and memory impairment caused by aging have not been reported, and the deep mechanism of antioxidant has not been elucidated.

Therefore, in this study, we investigated the effect of *Monarda didymaL.* essential oil and its main active ingredient thymol on improving the learning and memory ability of D-galactose-induced aging mice, in which Nrf2 and MAPK pathways might be involved.

## 2 Materials and methods

### 2.1 Reagents

*Monarda didymaL.* essential oil used in the experiment was originated from Heilongjiang Province, China, and cultivated by our team. Donepezil (Shanghai Yuanye Bio-Technology Co., Ltd., >98%, Shenyang, China); D-galactose (Beijing Dingguo Changsheng Biotech Co., Ltd., >98%, Beijing, China); SOD, MDA, and GSH-Px kits (Nanjing Jiancheng Bioengineering Institute, Nanjing, China); All antibodies for western blot were procured from Beijing Boao Biotechnology Co., Ltd.

### 2.2 Animals

Ninety SPF ICR mice (male; aged 6 weeks and weighing 18–22 g) were purchased from Changchun Yisi Experimental Animal Technology Co., Ltd. (license No. scxk-2018-0007). The mice were fed in separate cages at 20–24°C, in a humidity of 40%–60% and a 12 h day/night cycle, and with a free access to water and food. The experiments were started after the adaptive feeding in the laboratory for 3 days.

### 2.3 Analysis of components in Monarda didymaL. volatile oil by GC-MS

#### 2.3.1 Chromatographic conditions

1) Chromatographic column: DB-1 capillary column (0.25 mm × 60 m, 0.25 μm). 2) Carrier gas: nitrogen (99.999%). 3) Flow rate: 1 ml/min. 4) Sample inlet temperature: 250°C. 5) The shunt ratio is 40:1. 6) The injection volume is 2 μl. 7) Column temperature program: initial temperature maintained at 60°C for 4 min, and then increased to 150°C at 3°C/min for 10 min; Increase to 240°C at 10°C/min for 10 min.

#### 2.3.2 Mass spectrometric conditions

EI ion source temperature: 230°C; electron energy: 70 eV; connector temperature: 280°C; solvent delay: 7.5 min; scan range: m/z = 33–350; electron multiplier voltage: 2.4 kV.

### 2.4 Establishment of animal model and administration

Ninety male ICR mice were randomly divided into nine groups, control group, model group, donepezil group, and low-, medium- and high-dose *Monarda didymaL*. essential oil groups (oil-L, oil-M, and oil-H) and thymol groups (thymol-L, thymol-M, and thymol-H), 10 mice in each group. Except for those in control group, mice in the other groups were subcutaneously injected with 150 μg∙g-1 D-galactose and those in control group were injected subcutaneously with an equal volume of normal saline every day. At the same time, mice in all groups were intragastrically administered with the different agents once daily, in which mice in donepezil group were given 3 mg∙kg-1 donepezil, those in the low-, medium- and high-dose oil and thymol groups were given 20 mg∙kg-1, 40 mg∙kg-1, and 80 mg∙kg-1, mice in the control group and the model group were respectively given an equal volume of distilled water. All the mice were intragastrically given the different agents successively for 8 weeks, and the behavioral test was carried out 30 min after the administration 9 weeks 24 h after the behavioral test, hippocampal tissue samples of mice were taken for HE staining, AchE, SOD, GSH-Px activity, and MDA content, and Western blotting.

### 2.5 The behavioral test

#### 2.5.1 Morris water maze test

Morris water maze test consisted of three stages: visible platform stage, hidden platform stage and no platform stage. The experiment lasted for 6 days, and each animal was tested four times a day. The platform was placed in two different positions in the first two and the last two stages respectively. 3 days before the experiment, mice were sent to the water maze test room at fixed time every day for 4 h to get familiar with the environment. Stage 1 visibility period (1 day): Put platform at first as the center, the mice from the platform of the contralateral quadrants starting point for the wall, water free inquiry 60 s, find the platform system records mice swimming time (incubation period) and speed, if more than 60 s didn’t find the platform (record the incubation period of 60 s), and guide to the platform in mice after 15 s rest for the next test, each time interval 20 min. The second stage of hidden plateau (4 days): The mice were fixed 1 cm below the horizontal plane in the same position, and the mice were launched from any of the four starting points on the pool wall. The time (incubation period) and average speed of finding the platform of the mice were recorded. Go in a different sequence four times a day. The third stage no platform phase (1 day): The platform was evacuated, the target platform position was marked on the computer screen, and the mice were put into water at a position far from the target platform. The times of crossing the platform and the platform quadrant retention time of the mice were recorded within 60 s.

#### 2.5.2 Step-through test

The mice were placed in the test environment to adapt for 5 min, and then put into the bright box. After 10 s, the door of the bright and dark box was opened. After the mice fully entered the dark box, the door was closed immediately and an electric shock was given, which lasted for 1–2 s for multiple times within 5 min. After 24 h, the mice were re-placed in the bright box, and the door of the bright and dark box was opened. The time (incubation period) for the mice to enter the dark box for the first time and the times of entering and exiting within 5 min were observed and recorded, and the memory ability of the mice was evaluated.

### 2.6 Hematoxylin-eosin staining

Upon anesthesia, the mice were perfused transcardially with ice-cold saline solution followed by 4% paraformaldehyde. Brain tissues were immediately isolated and fixed in 4% paraformaldehyde solution for 24 h. After routine tissue processing, tissues were stained with hematoxylin-eosin to capture the shape of neurons. Images were taken with an optical microscope.

### 2.7 Detection of AchE, SOD, MDA, and GSH-Px in the hippocampus of mice

The hippocampal tissue samples of each group were homogenized, fully lysed with high-efficiency RIPA lysis buffer, centrifuged at 3500 r/min for 15 min, and the supernatant was collected. The oxidative stress indicators (GSH-Px, MDA content, and SOD activity) and AchE activity in the tissues were detected according to the operation steps of the kit manual.

### 2.8 Detection of Nrf2 and MAPK pathways related protein expression by western blot assay

Homogenates were taken from each group, RIPA Buffer lysate was added, centrifuged, and supernatant was collected. Total protein was extracted with total protein extraction kit, and protein concentration was measured by BCA method. After that, the protein samples were loaded, electrophoresis, membrane transfer and sealed. Primary antibodies P38, P-P38, ERK1/2, P-ERK1/2, Nrf2, HO-1, SOD2, and NQO-1 were dropped and incubated overnight at 4°C. Secondary antibodies were dropped and incubated for 2 h on a shaking bed. TBST was cleaned and ECL luminescence solution was added for imaging. GAPDH was used as internal reference.

### 2.9 Statistical analysis

The data were expressed as mean ± *s*. SPSS21.0 statistical software was used for the one-way ANOVA, and *t*-test was used to compare the results between groups, and *p* < 0.05 or *p* < 0.01 was considered to be statistically significant.

## 3 Results

### 3.1 Analysis of Monarda didymaL. essential oil composition

The components of *Monarda didymaL.* essential oil was analyzed by GC-MS method, which mainly included thymol, 2-platycladine, β-myrcene, pinene, terpene, cymene, terpinene, and δ-3-carene. Among them, thymol accounted for the largest amount, about 63.8% (Table 1).

**TABLE 1:** Composition of *Monarda didymaL.* essential oil.

Peak	Compounds	Peak area (%)	Retention time (min)
1	b-Thugene	3.62	6.51
2	α-Pinene	0.55%	6.67
3	Myrcene	1.63%	7.77
4	Terpinolene	2.89%	8.54
5	o-Cymene	11.0%	8.76
6	γ-Terpinene	16.6%	9.79
7	Thymol	63.8%	17.86

### 3.2 Effect of Monarda didymaL. essential oil and thymol on the performance of mice in morris water maze test

**FIGURE 1:**
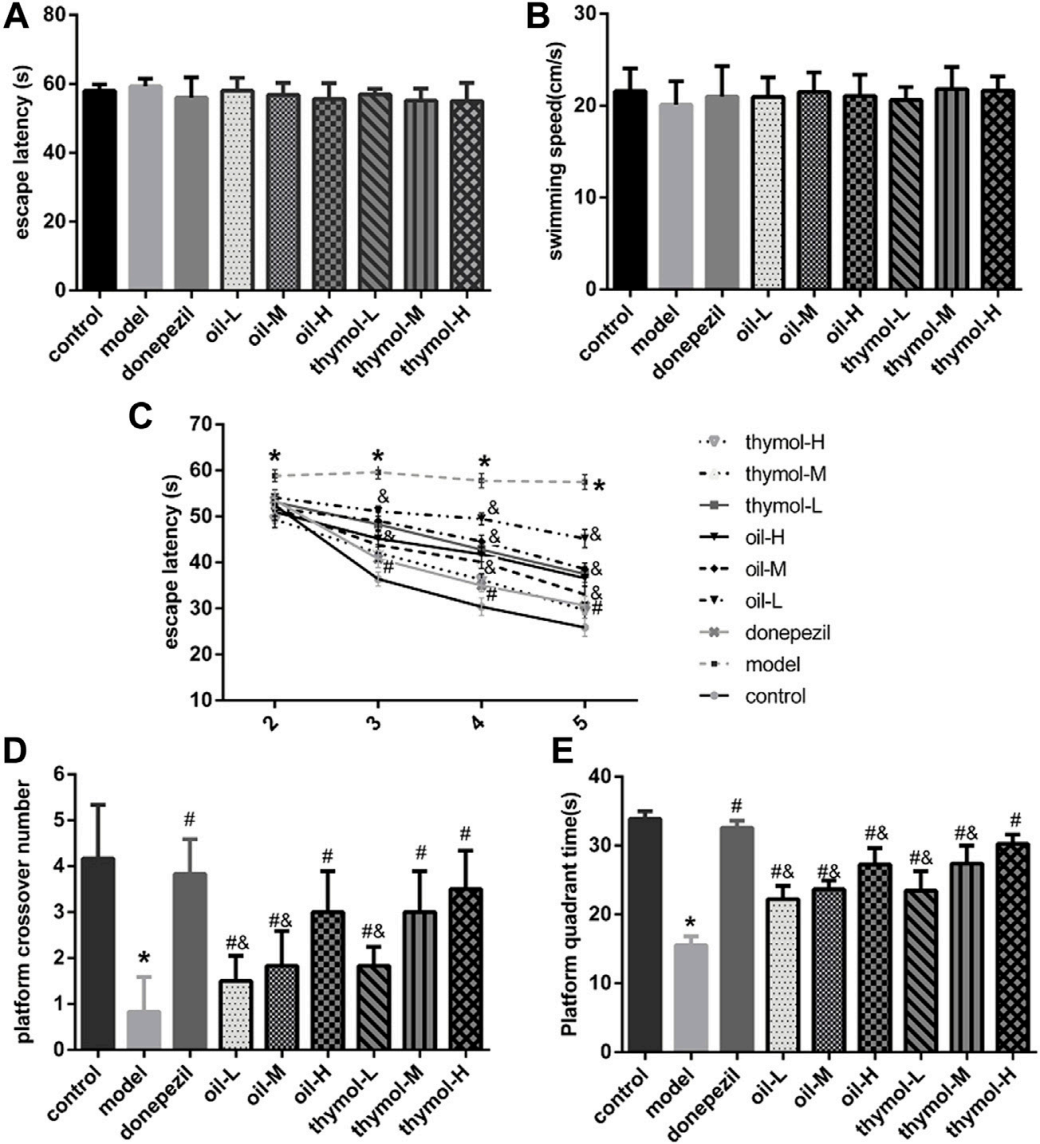
Comparison of water maze test results of mice in each group. **(A)** Comparison of escape latency of mice in plateau stage; **(B)** Comparison of average swimming speed of mice in the plateau stage; **(C)** Comparative analysis of escape latency of mice in hiding plateau stage; **(D)** Comparison of the number of times of mice crossing the platform in non-platform stage; **(E)** Comparison of platform quadrant activity time between groups in non-platform phase; Compared with control group *p < 0.05; Compared with model group #*p* < 0.05; compared with donepezil group &*p* < 0.05 *n* = 10.

The change of learning and memory ability of animals may be reflected in the detection of their behavior function, so we first used Morris water maze method to evaluate the learning and memory abilities of mice. The effects of *Monarda didymaL.* essential oil and thymol on learning and memory abilities were shown in Figure 1. There was no difference in the first day latency of mice among groups (*p* < 0.05), and the average speed of movement was not statistically different (*p* < 0.05) (Figures 1A,B), which indicated that all the mice in this experiment performed normally and could be used for subsequent experiments.

The results of positioning navigation test (Figure 1C) showed that the latency of mice in each group decreased to different degrees with the increase of training days after platform hiding. Compared with control group, latency of model group was significantly higher (*p* < 0.05); Compared with model group, the latency of mice in donepezil, oil and thymol groups decreased significantly, and the higher the dose of oil and thymol, the lower the latency. (*p* < 0.05). Compared with donepezil group, the latency of mice in oil-L, thymol-L, and thymol-M groups increased (*p* < 0.05), but there was no difference between thymol-H and Donepezil groups (*p* < 0.05).

The results of the space exploration test showed that (Figures 1D,E) after the platform was removed, the number of mice crossing the platform and the activity time in the platform quadrant in the model group were significantly lower than those in the control group (*p* < 0.05). Compared to the model group, donepezil, oil and thymol groups increased significantly (*p* < 0.05), and increased in a dose-dependent manner in oil and thymol groups. The activity time and the numbers of crossing in the platform quadrant were close between thymol-H group and donepezil groups. However, the durations of locomotor activity and the times of crossing over the platform in the quadrant of the platform between oil-H and thymol-M groups had similar effects without significant difference (*p* < 0.05).

### 3.3 Neuroprotective effects of Monarda didymaL. essential oil and thymol on a D-galactose-induced mice model in step-through test

**FIGURE 2:**
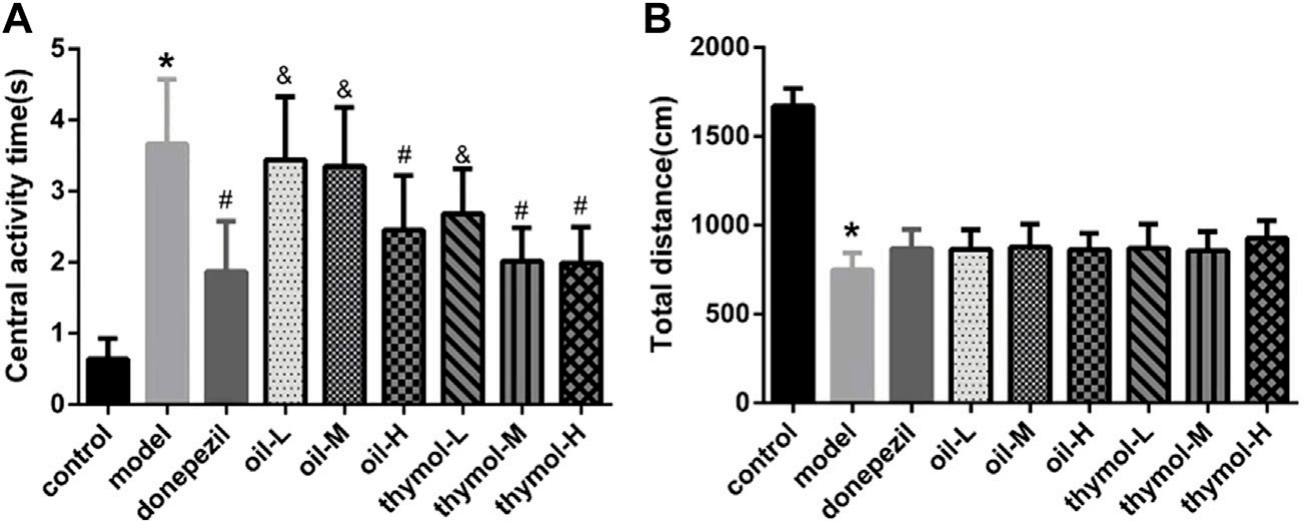
Comparison of step-through test of mice in each group. **(A)** Comparison of escape latency; **(B)** Comparison of error times; Compared with control group *p < 0.05; compared with model group #*p* < 0.05; compared with donepezil group &*p* < 0.05 *n* = 10.

The results of step-through test (Figure 2) showed that compared with the control group, the escape latency of the model group reduced significantly, and the number of errors increased (*p* < 0.05). Compared with the model group, the escape latency was prolonged and error times were reduced in the donepezil group. The escape latency was prolonged and error times were reduced in the oil and thymol groups in a dose-dependent manner to different extents. In the escape latency test, the effects of thymol-H and donepezil groups were similar without significant difference (*p* < 0.05), while the oil-H group was higher than that of thymol-M group (*p* < 05). And in the number of errors, there was also no significant difference between thymol-H and donepezil groups, oil-H and thymol-M groups (*p* < 0.05).

### 3.4 Effects of Monarda didymaL. essential oil and thymol on histopathological changes in the hippocampus

**FIGURE 3:**
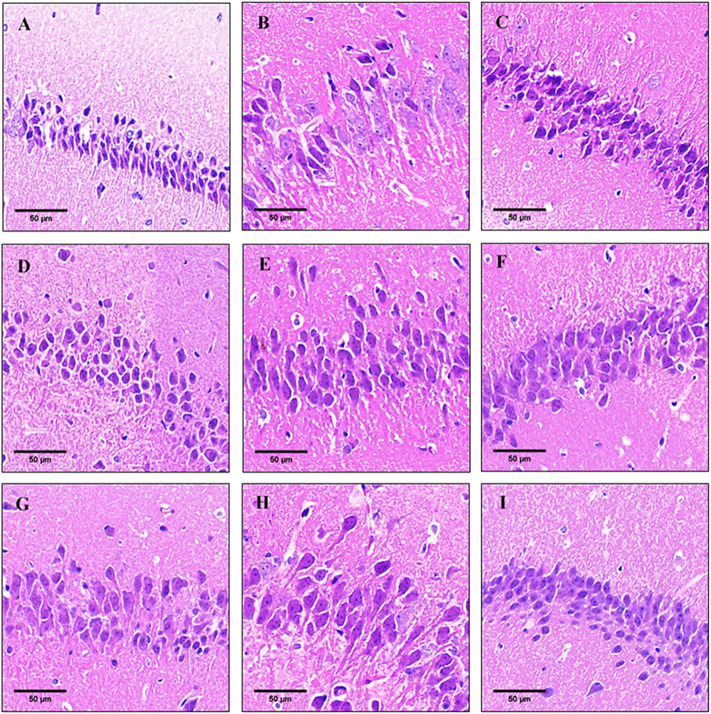
Pathological observation in hippocampal tissues of mice in each group (HE staining). **(A)** Control group; **(B)** model group; **(C)** donepezil group; **(D)** oil-L group; **(E)** oil-M group; **(F)** oil-H group; **(G)** thymol-L group; **(H)** thymol-M group; **(I)** thymol-H group. The scale is 50 μm.

The pathology results showed that changes in the histopathology features of the hippocampus sections as observed by hematoxylin and eosin (HE) staining (Figure 3). The neurons in the hippocampus of the control group were clearly stained, the cells were arranged closely and orderly, and the nuclei and cytoplasm were clearly visible (Figure 3A). Compared with the control group, the pyramidal neurons in the hippocampus of the model group had loose structure and disorderly arrangement, and the normal neurons were significantly reduced, with deepening staining and different cell morphology (Figure 3B). The results showed that long-term injection of D-galactose caused brain damage, neuron damage or loss in mice.

After treated with thymol and oil, the neuronal cell structure tended to be compact, the cells were arranged neatly, and normal neurons were increased with the increase of the dose. Oil-H and thymol-M groups showed no significant differences in other indicators except for slightly loose neuron arrangement, as compared with the donepezil group. Among them, the condition in the thymol-H group was significantly improved after treatment, and the effect was basically the same as that in the donepezil group (Figures 3C–I). These results indicated that oil and thymol had a protective effect on D-gal-induced hippocampal damage in a dose-dependent manner.

### 3.5 Effects of Monarda didymaL. essential oil and thymol on the serum SOD, GSH-Px, MDA, and AchE in mice

*In vivo* antioxidant experiments, the body’s ability to resist oxidative damage is generally reflected by the determination of the activities of SOD, GSH-Px, and MDA contents (Öztürk-Ürek et al., 2001; Liu et al., 2011). The change of AchE activity often leads to learning and memory dysfunction (Ruan et al., 2014). The activities of MDA, GSH-Px, SOD, and AchE in the hippocampus of each groups were shown in Table 2. Compared with the control group, the activities of SOD and GSH-Px in model group decreased significantly, while MDA increased significantly, indicating that the oxidation index and AchE activity were increased under the action of D-galactose. The results showed that the model successfully induced senescence (*p* < 0.05). Compared with model group, the activities of SOD and GSH-Px in oil and thymol groups increased in a dose-dependent manner, while the activities of AchE and MDA decreased in a dose-dependent manner. There was no significant difference between oil-H, thymol-H and donepezil groups. It was suggested that oil and thymol could effectively improve the oxidative stress response of damaged hippocampal tissue to some extent and played an anti-aging role.

**TABLE 2:** Comparison of oxidative stress indexes and AchE activity in each group (*n* = 5).

Group	SOD (U/mg)	GSH-Px (U/mg)	MDA (nmol/mg)	AchE (U/mg)
control	276.33 ± 12.89	236.78 ± 15.53	6.52 ± 0.80	0.41 ± 0.01
model	132.28 ± 6.77^*^	105.85 ± 9.36^*^	18.35 ± 1.89^*^	0.58 ± 0.08^*^
donepezil	271.45 ± 13.49^#^	229.57 ± 12.64^#^	6.24 ± 1.15^#^	0.42 ± 0.02^#^
oil-L	145.46 ± 8.93^#&^	137.62 ± 8.69^#&^	17.65 ± 2.03^#&^	0.54 ± 0.03^&^
oil-M	189.35 ± 11.28^#&^	159.35 ± 9.04^#&^	12.49 ± 1.25^#&^	0.50 ± 0.03^#&^
oil-H	256.23 ± 14.71^#^	210.81 ± 12.37^#^	6.89 ± 0.95^#^	0.45 ± 0.03^#^
thymol-L	174.52 ± 9.05^#&^	147.38 ± 8.66^#&^	14.60 ± 1.11^#&^	0.52 ± 0.03^#&^
thymol-M	234.64 ± 10.48^#&^	185.15 ± 9.34^#&^	10.36 ± 1.05^#&^	0.46 ± 0.02^#&^
thymol-H	263.18 ± 12.54^#^	227.04 ± 13.67^#^	5.97 ± 0.50^#^	0.41 ± 0.02^#^

Note: compared with control *p < 0.05; compared with model #*p* < 0.05; compared with donepezil and *p* < 0.05.

### 3.6 Effects of Monarda didymaL. essential oil and thymol on related proteins in the Nrf2 and MAPK pathways

**FIGURE 4:**
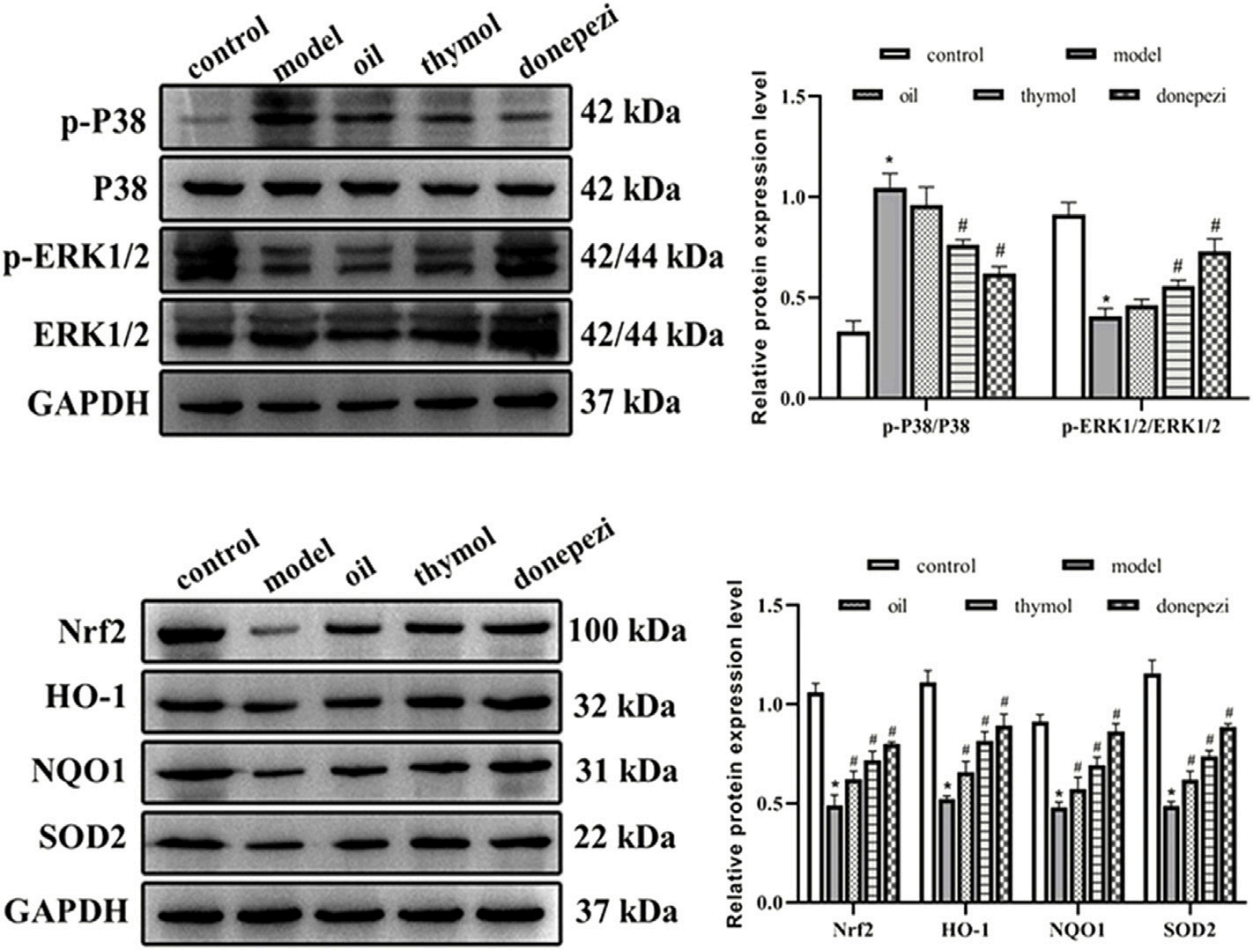
Comparison of related proteins expression levels in each group. Compared with control group *p < 0.05; Compared with model group #^p^<0.05; compared with donepezil group &*p* < 0.05 *n* = 10.

Nrf2 pathway is the first line for cells to defense against ROS, and regulate cell metabolism through a series of downstream reactions, while MAPK is recognized as an important signaling pathway mediating cell growth and proliferation, both of which are involved in the mechanism of various brain nerve cell injury related neurodegenerative diseases (Zhou et al., 2019). Therefore, the expressions of Nrf2 and MAPK pathways related proteins in each group were detected in this study. The results (Figure 4) showed that p-P38/P38 ratios in model group increased significantly compared with control group. The expression level of P-ERK/ERK decreased significantly (*p* < 0.05). Compared with model group, p-P38/P38 in Donepezil, oil and thymol groups significantly decreased, and p-ERK/ERK expression level significantly increased (*p* < 0.05). Compared with the control group, the expression levels of Nrf2, HO-1, SOD2, and NQO1 in the model group were relatively lower (*p* < 0.05), while donepezil, oil and thymol treatments significantly increased the levels of Nrf2, HO-1, SOD2, and NQO1. These data suggested that Nrf2 and MAPK pathways may be involved in the effects of *Monarda didymaL.* essential oil and thymol on the neural function of aging mice.

## 4 Discussion

Oxidative stress theory is one of the mainstream theories on the pathogenesis of neurodegenerative diseases, which is closely related to the occurrence and development of typical degenerative diseases such as AD, Parkinson’s disease (PD) and amyotrophic lateral sclerosis (ALS). Oxidative stress refers to the stimulation of the body, the excessive production of high-activity molecules, such as reactive oxygen species (ROS) and reactive nitrogen radicals (RNS) in the body, resulting in the imbalance of antioxidant protection system. ROS/RNS exceed the clearance capacity of cell antioxidant system, thus causing oxidative damage to tissues and cells. The hypoxia effect in aging brain further promotes the production of ROS, aggravates oxidative stress damage, and accelerates the neurodegenerative disease process (Islam, 2016). Therefore, based on the mechanism of aging and aging related diseases, it is an important direction in the field of learning and memory function of geriatrics to explore anti-aging drugs to prevent or reduce learning and memory impairment continuously.

At present, most of the drugs mainly used in clinical treatment of neurodegenerative diseases can only partially relieve symptoms, or relieve cognitive, motor, and mental disorders in early and middle stage patients, and it is difficult to achieve satisfactory therapeutic effects. In recent years, with the development of traditional Chinese medicine, most scholars have found that natural active ingredients with antioxidant function may become the key basis for the treatment of neurodegenerative disease.

Essential oil is an oily liquid with volatile property extracted from plants, and have good antioxidant and anti-inflammatory effects proved by a large number of studies. It is widely used in cosmetics, food, health drugs, and nervous system related diseases. Studies have shown that the virgin olive oil and lemon volatile oil can reduce the AchE enzyme activity and MDA level in the hippocampus of mice with stress injury to a certain extent, increase the activity of SOD and GSH-Px, improve the learning and memory abilities of mice, and prevent AD (Falls et al., 2018; Tzekaki et al., 2021). The study of Boiangiu et al. (2020) found that compound essential oils [ingredients: Limonene (91.11%), followed by gamma - pine oil olefin (2.02%), beta myrcene (1.92%), beta pinene (1.76%), alpha pinene (1.01%), terpene (0.67%), linalool (0.55%), cymene (0.53%), and valerian (0.43%)] had strong antioxidant effect, which could effectively reduce the amnesia and oxidative stress damage of hippocampal tissue in rats, and improve the learning and memory abilities of model rats to a certain extent. In this study, GC-MS analysis showed that *Monarda didymaL.* essential oil contains thymol, 2- platycladine, β-myrcene, pinene, terpene, cymene, terpinene, and δ-3-carene, part of the components was the same as the above-mentioned compound essential oil components of boiangiu RS, and studies confirmed that *Monarda didymaL.* essential oil and its main component thymol had strong antioxidant effects, Capibaribe et al. (2019) found that thymol had neuroprotective effects on the central nervous, which could effectively improve behavioral function, reduce anxiety, restore short-term memory function, and improve depressive symptoms in animal models of depression. It was also reported that thymol improved learning and memory abilities of HFD (high fat diet)-induced cognitive dysfunction animal models, improved HFD-induced hippocampal Aβ deposition and tau hyper phosphorylation, and inhibited oxidative stress and inflammatory response in hippocampal tissues (Asadbegi et al., 2017). However, the studies on the *Monarda didymaL.* essential oil and its main component thymol in neurodegenerative diseases are relatively rare, and the protective effects on neurological function and learning and memory ability are not clear yet.

A large number of studies have proved that long-term injection of D-galactose can cause aging of the body, and this model is highly similar to natural aging of the organism. Therefore, D-galactose often used to make animal aging models (Xie et al., 2018; Doan et al., 2015; Li et al., 2021) in this study, a mouse model of aging was established by subcutaneous injection of D-galactose. Morris water maze test and biochemical indices were used to study the effects of menthol and thymol on learning and memory in mice. In Morris water maze test, compared with model group, latency of mice in each group reduced significantly after treatment, and the times of crossing the platform and the activity time in the platform quadrant increased. The changes of oil and thymol concentration groups were dose-related, and the changes of thymol were greater at the same dose. There was no statistical difference between the high-dose and donepezil groups. It was suggested that the essential oil and thymol could improve the learning and memory abilities of aging mice, and the effect was dose-dependent, and thymol had the better effect. The content of thymol in the high dose of essential oil was much lower than that in the medium dose, but the effect was similar, which reflected the synergistic effect of other components of essential oil and thymol, indicating that oil had more potential in treatment. In order to ensure the reliability of the experimental conclusions, we tested the platform response time and the number of errors in the step-through test of mice in each group. The results showed that compared with the model group, the platform response time and the number of errors in the platform response time decreased and the latency increased in the medication group. The number of dark avoidance errors increased, and the incubation period decreased significantly. The change of oil, thymol concentration groups was dose-related, and the change of high dose was the most significant, but the effect of thymol was better at equal dose. Moreover, there was no significant difference between the oil-H and thymol-M, which once again verified the above conclusion. These results indicated that the *Monarda didymaL.* essential oil. And thymol could effectively improve the learning, memory, autonomy and exploratory behavior of aging mice, and preliminarily verified the hypothesis that the *Monarda didymaL.* essential oil. Could effectively improve learning and memory abilities at the behavioral level.

The signaling of neuronal synapses in the hippocampus is closely related to learning and memory, so maintaining the structural integrity of neurons in the hippocampus is important for improving learning and memory deficits (Pirger et al., 2009). The results of the HE staining experiment showed that pyramidal neurons in the hippocampus region of aging mouse model had loose structure, sparse and disordered arrangement, and abnormal morphology, which was speculated to be caused by long-term chronic oxidative stress injury induced by D-galactose. After treatment, the rate of neuronal apoptosis and neuronal injury were reduced to different degrees, and the neuronal structure, sequence and cell morphology seemed to be reduced to different degrees. Among them, the effects of oil -H and thymol -M on the above indicators were similar to that in the donepezil group. Except for the slightly loose arrangement of neurons, there was no significant difference in other indicators, suggesting that the they had similar efficacy.

Oxidative stress can create an imbalance between the efficiency of antioxidant systems of enzymes (e.g., catalase, GSH-PX, and SOD) and non-enzymes (e.g., reduced GSH) (Borys et al., 2019; Lushchak, 2014). Abnormal redox reaction in the body can produce excessive free radicals such as ROS, which can attack intracellular biological macromolecules, including nucleic acids, protein and lipids. MDA is a product of oxidative toxicity that is produced by lipid peroxidation and causes cross-linking of nucleic acids, and excessive consumption of intracellular antioxidant enzymes. This series of changes can finally lead to characteristic neurodegenerative pathological changes such as aging and loss of neurons in the brain (Esposito, 2006; Devore et al., 2010; Buendia et al., 2015). GSH-Px is an important peroxidase in the body that can catalyze GSH to produce GSSG, thus protecting the structural and functional integrity of cell membrane. The contents of MDA and the activities of SOD and GSH-Px in the hippocampus of aging mice in each group were measured. The results showed that compared with the model group, the activities of SOD and GSH-px enzymes in the tissues of each groups increased after treatment, while the content of MDA decreased. The changes of the above indicators in the high dose group were more significant, and thymol had a better effect at the same dose. These results indicated that both *Monarda didymaL.* essential oil and thymol could improve the oxidative stress response in aging mice, and the effect was dose-dependent. Besides, the efficacy of oil-H was better than that of thymol-M, indicating that other components in the *Monarda didymaL.* essential oil except thymol also played a role in improving the oxidative stress response of aging mice.

MAPK pathway is an important signal transduction pathway in human cells, which widely exists in the central nervous system, transduces extracellular signal stimuli to intracellular or nuclear under physiological state, participates in synaptic transmission, neuronal remodeling, morphological differentiation as well as cell proliferation, apoptosis and other physiological processes, which is closely related to the occurrence of many diseases in the nervous system. Five MAPK signal transduction pathways have been identified in eukaryotic cells: extracellular signal-regulated protein kinase (ERKI/2 also known as P44/42MAPK), c-Jun N-terminal Kinase (JNK also known as SAPKI), p38 mitogen-activated protein kinase (P38, also known as SAPK2), ERK3/4 and extra Cellular signal-regulated kinase 5 (ERK5) subfamily, each subfamily in turn contains multiple subtypes (Kyriakis and Avruch., 2001; Saito., 2013). A large body of researches showed that exogenous oxidative stress could lead to the activation of intracellular MAPK pathway. ROS induced by hypoxia or ROS inducers was involved in the activation of p38 and phosphorylation of JNK (Niso-Santano et al., 2010; Li et al., 2012).

Nrf2 pathway is a typical signaling pathway in the processes of anti-oxidative stress and anti-aging. The activation of Nrf2 and its downstream signaling is one of the main means to mediate the intracellular antioxidant defense system, and the main action pathway by which multiple antioxidants, including natural compounds and synthetic products, exert antioxidant effects *in vitro* and *in vivo*. Studies showed that chrysin, apigenin, and chrysoeriol could exert cytoprotective effects against oxidative stress through upregulation of ERK phosphorylation driving Nrf2 nuclear translocation and increased the expression of its downstream protein HO-1 (Yang et al., 2015). Ginkgo extracts could activate phosphorylated ERK in mouse C2C12 myoblasts, leading to the expression of nuclear Nrf2 and downstream HO-1, whereas application of the ERK inhibitor TO98059 could significantly inhibit Nrf2 nuclear translocation and HO-1 upregulation (Innamorato et al., 2008). Other studies showed that the activation of ERK was involved in the regulation of synaptic stability and plasticity, and was beneficial to the maintenance of spatial learning and memory ability of animals (Hebert., 2002). In this study, we detected the change of Nrf2 and MAPK pathway, and the results showed that p-P38/p38 decreased to different degrees after different drug treatments, and the expression levels of p-ERK/ERK, Nrf2 HO-1, SOD2, and NQO-1 increased. Similar to many natural antioxidants, *Monarda didymaL.* essential oil could not only remove ROS, but also reduce the phosphorylation of p38 in cells, indicating that the regulation of MAPK signaling pathway might be involved in the neuroprotective effects of *Monarda didymaL.* essential oil and thymol. *Monarda didymaL.* essential oil and thymol further promoted the expression of Nrf2, HO-1, SOD2, and NQO-1 by promoting Nrf2 nuclear translocation, thus playing a protective role in anti-aging.

## 5 Conclusion

In conclusion, this study concluded that the *Monarda didymaL.* essential oil and its main component thymol could effectively improve the learning and memory abilities of mice. Thymol was the main effector, and other components in the *Monarda didymaL.* essential oil besides thymol also played a certain role. Its mechanism may be related to Nrf2 and MAPK pathways, but further study is needed to confirm this theory.

## Data Availability

The original contributions presented in the study are included in the article/supplementary material, further inquiries can be directed to the corresponding authors.
